# Genome‐wide methylation profiling of maternal cell‐free DNA using methylated DNA sequencing (MeD‐seq) indicates a placental and immune‐cell signature

**DOI:** 10.1111/eci.14363

**Published:** 2024-11-26

**Authors:** Marjolein M. van Vliet, Ruben G. Boers, Joachim B. Boers, Olivier J. M. Schäffers, Lotte E. van der Meeren, Régine P. M. Steegers‐Theunissen, Joost Gribnau, Sam Schoenmakers

**Affiliations:** ^1^ Department of Obstetrics and Gynaecology Erasmus MC Rotterdam The Netherlands; ^2^ Department of Developmental Biology Erasmus MC Rotterdam The Netherlands; ^3^ Department of Pathology Erasmus Medical Centre Rotterdam Rotterdam The Netherlands; ^4^ Department of Pathology Leiden University Medical Center Leiden The Netherlands

**Keywords:** cell‐free DNA, epigenetics, placenta, pregnancy

## Abstract

**Background:**

Placental‐originated cell‐free DNA (cfDNA) provides unique opportunities to study (epi)genetic placental programming remotely, but studies investigating the cfDNA methylome are scarce and usually technologically challenging. Methylated DNA sequencing (MeD‐seq) is well compatible with low cfDNA concentrations and has a high genome‐wide coverage. We therefore aim to investigate the feasibility of genome‐wide methylation profiling of first trimester maternal cfDNA using MeD‐seq, by identifying placental‐specific methylation marks in cfDNA.

**Methods:**

We collected cfDNA from nonpregnant controls (female *n* = 6, male *n* = 12) and pregnant women (*n* = 10), first trimester placentas (*n* = 10), and paired preconceptional and first trimester buffy coats (total *n* = 20). Differentially methylated regions (DMRs) were identified between pregnant and nonpregnant women. We investigated placental‐specific markers in maternal cfDNA, including *RASSF1* promoter and Y‐chromosomal methylation, and studied overlap with placental and buffy coat DNA methylation.

**Results:**

We identified 436 DMRs between cfDNA from pregnant and nonpregnant women, which were validated using male cfDNA. *RASSF1* promoter methylation was higher in maternal cfDNA (fold change 2.87, unpaired *t*‐test *p* < .0001). Differential methylation of Y‐chromosomal sequences could determine fetal sex. DMRs in maternal cfDNA showed large overlap with DNA methylation of these regions in placentas and buffy coats. Sixteen DMRs in maternal cfDNA were specifically found only in placentas. These novel potential placental‐specific DMRs were more prominent than *RASSF1*.

**Conclusions:**

MeD‐seq can detect (novel) genome‐wide placental DNA methylation marks and determine fetal sex in maternal cfDNA. Our results indicate a placental and immune‐cell contribution to the pregnancy‐specific cfDNA methylation signature. This study supports future research into maternal cfDNA methylation.

## Graphical abstract

Studies investigating the maternal cell‐free DNA (cfDNA) methylome are scarce. We identified 436 autosomal differentially methylated regions (DMRs) between cfDNA from pregnant and nonpregnant women, using methylated DNA sequencing (MeD‐seq). Y‐chromosomal methylation could determine fetal sex, we show hypermethylation of the placental‐marker *RASSF1*, and identify 16 novel placental‐specific markers in maternal cfDNA including DMRs related to *TMEM240*, *DHRS3* and *PCMTD2*. Additionally, DMRs largely overlap with DNA methylation of buffy coats indicating placental and immune‐cell contributions to the pregnancy‐specific cfDNA methylation signature.

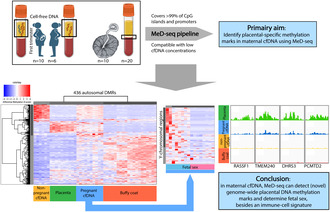

## 1 BACKGROUND

A well‐functioning placenta is essential for healthy fetal development, but the placenta is largely inaccessible during gestation. The direct study of placental tissue necessitates invasive procedures,^1^ or postpartum placental examination. Therefore, less invasive techniques to study placental development throughout gestation are warranted.

Turnover and shedding of placental cells result in the release of placental‐originated cell‐free DNA (cfDNA) in the maternal blood stream. Maternal plasma‐derived cfDNA is widely used to perform noninvasive prenatal testing (NIPT), enabling screening for chromosomal abnormalities.^2^ However, delving into the epigenetic landscape of maternal cfDNA could allow for applications beyond current practice.^3^ DNA methylation is an important epigenetic mechanism playing a crucial role in placental growth, development and function by regulating gene‐expression.^4^, ^5^, ^6^ Differences in placental DNA methylation have been associated with gestational age and adverse obstetrical outcomes including pre‐eclampsia.^7^, ^8^, ^9^ Maternal plasma‐derived cfDNA could facilitate the noninvasive study of placental DNA methylation in health and disease already from the first trimester onwards.

A major challenge in using maternal cfDNA as a proxy for placental DNA is the relatively low contribution of placental‐derived cfDNA to total maternal cfDNA. The largest contributor to maternal cfDNA is the maternal haematopoietic system, with a placental‐derived fraction of roughly 10% at the end of first trimester.^10^, ^11^, ^12^ However, since DNA methylation is highly cell type specific, cfDNA methylation profiles can aid in determining their tissue of origin.^4^, ^13^

Previous studies identifying placental‐specific DNA methylation markers mostly focused on methylation differences on chromosomes 13, 18 and 21, which have been shown to be potentially useful in screening for chromosomal abnormalities,^14^, ^15^, ^16^, ^17^, ^18^, ^19^, ^20^, ^21^, ^22^, ^23^ and/or focused on methylation differences between placental tissues and maternal whole blood.^24^, ^25^, ^26^ Nonetheless, our understanding of the genome‐wide impact of pregnancy directly on the cfDNA methylome, and how this reflects changes in maternal and placental tissues is limited. The scarce studies on cfDNA are mostly performed with whole genome bisulfite sequencing,^27^, ^28^, ^29^ which is costly and technologically challenging due to degradation of already low DNA levels in cfDNA samples, or achieved a limited genome‐wide coverage.^30^ At the same time, recent studies support the use of the cfDNA methylome as an early predictive marker for pre‐eclampsia development.^31^, ^32^, ^33^ This emphasizes the need to further explore the epigenetic landscape of cfDNA already during the early stages of pregnancy, ideally using less costly methods while ensuring a high genome‐wide coverage.

We therefore used Methylated DNA sequencing (MeD‐seq) to investigate its feasibility to study the maternal cfDNA methylome. MeD‐seq is compatible with cfDNA and covers >50% of genome‐wide cytosine‐guanine dinucleotides (CpGs), detecting DNA methylation at >99% of all CpG islands and promoters.^34^, ^35^ We aim to investigate the impact of pregnancy on the first trimester maternal cfDNA methylome, and explore the placental and haematopoietic origin of identified differences. We further aim to identify placental‐specific methylation marks in cfDNA. As proof‐of‐principle, we show the possibility to determine fetal sex in maternal cfDNA based on Y‐chromosomal methylation marks.

## 2 METHODS

### 2.1 Study design

We included pregnant women who participated in the Rotterdam Periconception Cohort (Predict study) at the Erasmus Medical Centre, Rotterdam. The Predict study is an ongoing prospective cohort study where women with a singleton pregnancy are recruited before 10 weeks of gestation and longitudinally followed throughout pregnancy.^36^, ^37^ Blood samples for cfDNA isolation were obtained around 11 weeks of gestation between November 2022 and March 2023.

Blood samples for cfDNA isolation from nonpregnant controls were previously obtained for research purposes from anonymous, healthy blood donors (HBDs) enrolled via the Dutch National blood bank (Sanquin). Additionally, we prospectively collected first trimester (around 9–12 weeks of gestation) placental tissues after elective surgical abortions at a Dutch abortion clinic between July and August 2023. All pregnancies had to be without known congenital abnormalities. Lastly, maternal paired buffy coats were collected from women who participated within the Predict study both before pregnancy (preconception) as in the first trimester of a subsequent pregnancy.

Both the Predict study (MEC‐2004‐227) and the research protocol to collect placental tissues (MEC‐2022‐0788) were approved by the Medical Ethics Committee of the Erasmus Medical Centre. All participants provided written informed consent.

### 2.2 Processing of samples

Blood samples for cfDNA isolation were collected in CellSave preservative tubes. Plasma was subsequently isolated by two centrifugation steps (10 min at 1711*g*—2000 *g* followed by 10 min 12,000 *g*—16,000 *g*).^38^ Blood samples for DNA isolation of buffy coats were collected in EDTA tubes. Buffy coats were isolated after centrifugation of the blood sample (10 min at 2000*g*). Plasma and buffy coats were stored at −80°C prior to (cf)DNA isolation. Placental tissues were stored in buffered 4% formaldehyde solution.

### 2.3 (cf)DNA isolation

cfDNA was isolated from 4 mL of plasma using the QIAamp Circulating Nucleic Acid Kit. DNA extraction and isolation from buffy coats were performed using the TECAN freedom EVO robot combined with the Promega ReliaPrep™ Large Volume HT gDNA Isolation System. Placental samples were formalin‐fixed and full thickness biopsies were taken followed by embedding in formalin‐fixed paraffin‐embedded (FFPE) blocks. Four to six slides of 4–6 μm were cut. One slide per sample was stained using haematoxylin and eosin (H&E) and placental parenchyma was evaluated by a perinatal pathologist (LEM). DNA was extracted and isolated from subsequent slides with normal first trimester placental parenchyma using the QIAamp DSP DNA FFPE Tissue kit according to the manufacturer's protocol.

### 2.4 MeD‐seq assay

MeD‐seq assays were essentially performed as previously described.^34^, ^35^, ^39^ First, genomic DNA and plasma‐derived cfDNA were digested with the methylation‐dependent restriction enzyme LpnPI (New England Biolabs, Ipswich, MA) generating 32 bp DNA fragments containing the methylated CpG in the middle. The TruPLEX DNAseq 96D kit (Rubicon Genomics, Takara Bio Europe, Saint‐Germain‐en‐Laye, France) was used to prep samples for sequencing and samples were purified on a Pippin HT system with 3% agarose gel cassettes (Sage Science, Beverly, MA). Samples were sequenced on the Illumina NextSeq2000 platform. Samples were first sequenced until ±2 M reads, and continued to a total of ±20 M reads if at least 20% of reads passed the LpnPI filter (see Section 2.5).

### 2.5 Data processing and analysis

Dual indexed samples were demultiplexed using bcl2fastq software (Illumina). Differentially methylated regions (DMRs) were detected using a previously established bioinformatics pipeline.^34^, ^35^, ^39^ Custom python scripts were used to process acquired DNA methylation profiles. Raw fastq files were subjected to Illumina adaptor trimming and reads were filtered based on LpnPI restriction site occurrence between 13 and 17 bp from either the 5′ or 3′ end of the read to filter out DNA fragments that were methylated. Reads that passed the filter were mapped to hg38 using bowtie2 and BAM files were generated using SAMtools version 0.1.19. Genome‐wide individual LpnPI site scores were used to generate read count scores for: transcription start sites (TSS, 1 kb before and 1 kb after), CpG‐islands and gene bodies (1 kb after TSS till TES). Gene and CpG‐island annotations were downloaded from ENSEMBL (Homo_sapiens_hg38.GRCh38.79.gtf, www.ensembl.org).

Additionally, a sliding window technique was used to detect DMRs and the chi‐squared test on read counts was used for statistical testing. A Bonferroni corrected *p*‐value ≤.05 was considered statistically significant. Z‐score transformation of the read count data was applied for unsupervised hierarchical clustering analyses.

DMRs were identified between cfDNA from pregnant and nonpregnant women. cfDNA from male HBDs were used as validation. Y‐chromosomal methylated DNA sequences were identified between cfDNA from male and female HBDs and subsequently applied to maternal cfDNA to determine fetal sex. Methylation of the *RASSF1* promoter, known to be hypermethylated specifically in placental tissues,^40^ was compared between cfDNA from pregnant and nonpregnant women using an unpaired *t*‐test standardized for number of reads per million (rpm). For DMRs identified in cfDNA, overlap with first trimester placental tissues and buffy coats was studied to explore tissue of origin of identified DMRs. To determine the presence or absence of cfDNA methylation signatures associated specifically with pregnant or with nonpregnant women, receiver operating characteristic (ROC) curves were calculated for each sample for each individual DMR. ROC curves of reference sets of pregnant women compared to nonpregnant women were used to calculate the optimal threshold (using the ‘scikit‐learn’ package Python) for each individual DMR. Samples above the threshold scored ‘1’, samples under the threshold scored ‘0’, leading to a binary score for each DMR. To investigate overlap of identified DMRs in cfDNA with placental tissues and with buffy coats, a cut‐off value of ≥80% of samples scoring ‘1’ or ‘0’ was used for hypermethylated and hypomethylated DMRs, respectively.

## 3 RESULTS

An overview of used samples and their technical performance is provided in Table S1.

### 3.1 DMRs between pregnant and nonpregnant cfDNA

**FIGURE 1:**
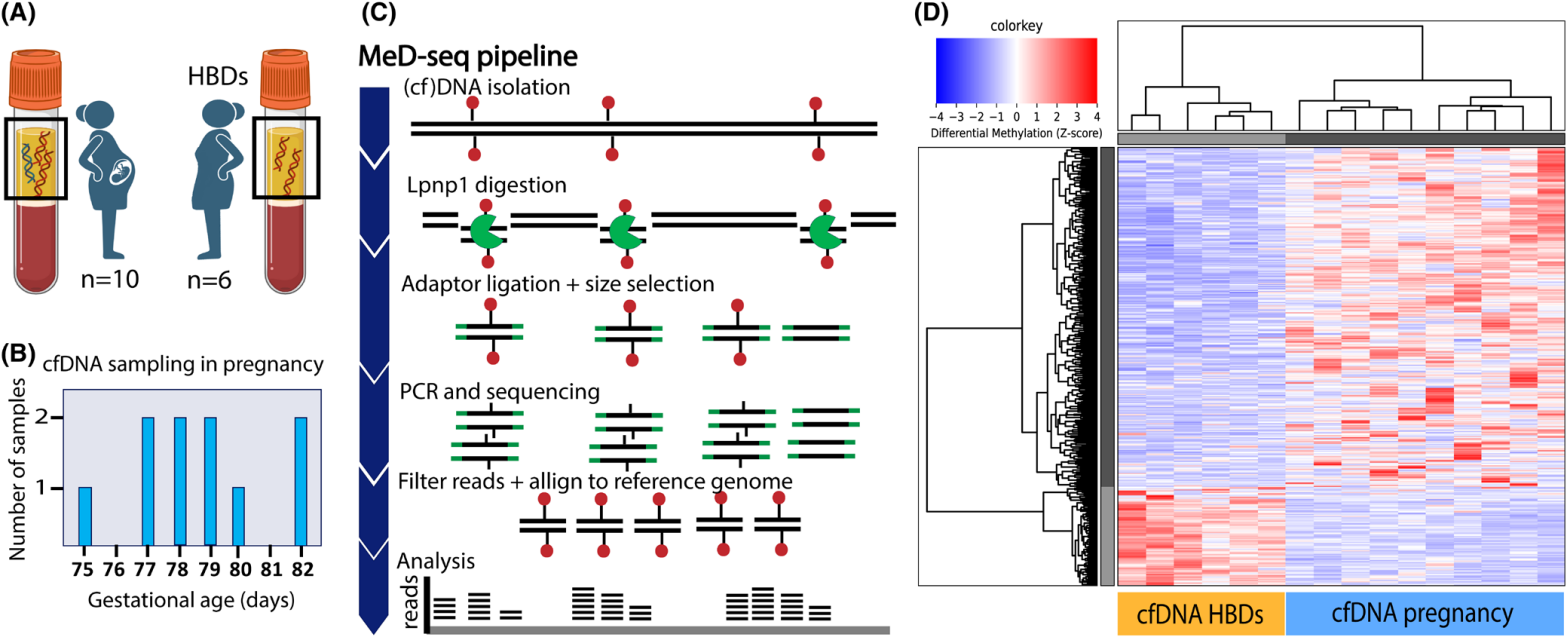
Identification of DMRs between cfDNA from pregnant (first trimester) and nonpregnant women (HBDs). (A) Study population consists of pregnant women at the end of the first trimester and nonpregnant women. cfDNA in plasma from pregnant women partly originates from the placenta. (B) Gestational age at time of cfDNA sampling in pregnancy. (C) Schematic overview of the MeD‐seq pipeline. (cf)DNA is digested by LpnPI which recognizes methylated CpGs and cuts the DNA 16 bp up‐and downstream leading to 32 bp fragments. This is followed by adaptor ligation, size selection of cfDNA fragments (32 bp), amplification and sequencing using the Illumina NextSeq2000 platform. Reads are filtered based on a central methylated CpG and aligned to a reference genome (hg38) prior to analysis. (D) Heatmap showing an unsupervised hierarchical clustering using autosomal DMRs with a Fold Change ≥2 between cfDNA from pregnant women in first trimester and nonpregnant women (HBDs) showing clear separation between the two groups. Z‐scores on normalized MeD‐seq data are used to visualize the data, red represents hypermethylation, blue represents hypomethylation.

To identify differences in cfDNA methylation related to pregnancy, we compared cfDNA from pregnant (*n* = 10) and nonpregnant women (HBDs, *n* = 6) (Figure 1A). For pregnant women, baseline characteristics are depicted in Table 1, and cfDNA was collected between 10 + 5 and 11 + 5 weeks of gestation (Figure 1B). Using MeD‐seq (Figure 1C), we identified 436 autosomal DMRs with a fold change (FC) ≥2, of which 338 (77.5%) were hypermethylated in pregnant women (Table S2). Unsupervised hierarchical clustering using identified DMRs shows clear clusters between pregnant and nonpregnant women (Figure 1D). As a validation, we show large overlap of male cfDNA (*n* = 12) with nonpregnant women showing robustness of our results (Figure S1).

**TABLE 1:** Baseline characteristics of pregnant women with cfDNA samples (*n* = 10).

Maternal age (years)	32.4 (30.7–34.3)
BMI at study entry	25.1 (22.8–26.8)
Country of birth	
Dutch	8 (80%)
Non‐Dutch	2 (20%)
Nulliparous	6 (60%)
Smoking during pregnancy	1 (10%)
Mode of conception	
Spontaneous	4 (40%)
IVF/ICSI	6 (60%)
Folic acid supplement use	10 (100%)
Started preconception	9 (90%)
Male fetus	5 (50%)

*Note*: Categorical variables are described using absolute numbers and percentages (%). Continuous variables are described using medians and interquartile ranges (IQR).

Abbreviation: IVF/ICSI, in vitro fertilization/intracytoplasmic sperm injection.

### 3.2 Placental origin of methylation profiles in cfDNA from pregnant women

**FIGURE 2:**
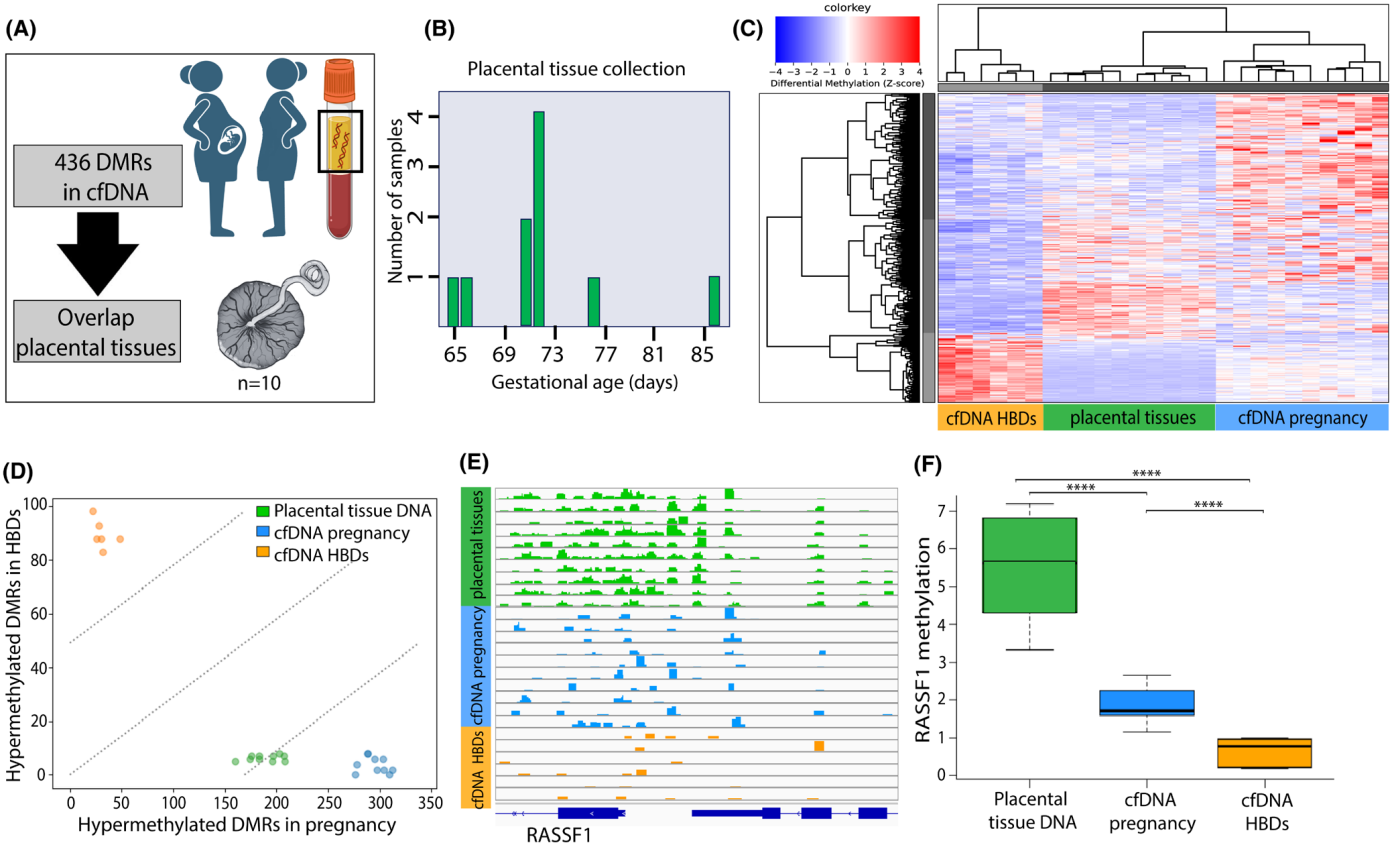
Placental origin of methylation profiles in cfDNA from pregnant women. (A) DMRs were identified in cfDNA between nonpregnant women (HBDs) and pregnant women in the first trimester. For identified DMRs, overlap with first trimester placental tissue biopsies is studied. (B) Gestational age at time of placental tissue collection. (C) Heatmap visualizing unsupervised hierarchical clustering of first trimester placental tissue biopsies between cfDNA from pregnant women and nonpregnant women (HBDs). Selected autosomal DMRs were identified between pregnant and nonpregnant women (HBDs). Red represents hypermethylation, blue represents hypomethylation. (D) After generating a cumulative methylation score, most DMRs hypermethylated in cfDNA from pregnant women are also hypermethylated in first trimester placental tissue biopsies (x‐axis). DMRs specifically hypermethylated in cfDNA from nonpregnant women (HBDs) are not hypermethylated in first trimester placental tissue biopsies (y‐axis). Each dot represents one sample. (E, F) Gene‐tracks and boxplots show hypermethylation of the RASSF1 promoter in placental tissue biopsies, and higher methylation of the RASSF1 promoter in cfDNA from pregnant women compared to cfDNA from nonpregnant women (HBDs) (*t*‐test *p* < .0001). *In our genome‐wide analysis, the difference in RASSF1 promoter methylation between cfDNA from pregnant and nonpregnant women did not remain statistically different after Bonferroni correction.

To explore which DMRs in maternal cfDNA could be of placental origin, we compared DMRs identified in cfDNA with MeD‐seq data from first trimester placental tissues (*n* = 10) (Figure 2A). Placental tissues were collected between 9 + 2 and 12 + 2 weeks of gestation (Figure 2B, Table S1). Unsupervised hierarchical clustering analysis shows that first trimester placental tissues cluster between cfDNA from pregnant and nonpregnant women (Figure 2C). Based on our cumulative methylation score, the majority of DMRs hypermethylated in cfDNA from pregnant women were in general also detected in placental tissues, while DMRs hypermethylated in cfDNA from nonpregnant women were not found in placental tissues (Figure 2D, Table S3). More strictly, of 338 DMRs hypermethylated and 98 DMRs hypomethylated in cfDNA from pregnant women, 143 (42.3%) and 88 (89.8%) were, respectively, consistently also hypermethylated or hypomethylated in ≥80% of placental tissues (Table S3). DMRs that are hyper‐or hypomethylated in both maternal cfDNA and placental tissues, as compared to cfDNA from nonpregnant women could indicate a placental‐origin of these DMRs in maternal cfDNA.

A well‐studied placental‐specific DNA methylation mark is hypermethylation of the *RASSF1* promoter.^41^ As expected, the *RASSF1* promoter displayed a higher methylation level in first trimester placental tissues as compared to cfDNA from pregnant or nonpregnant women (Figure 2E,F, Table S4). Moreover, the *RASSF1* promoter displayed a higher methylation level in cfDNA from pregnant as compared to nonpregnant women (FC 2.87, unpaired *t*‐test *p* < .0001) (Figure 2E,F, Table S4), although this did not remain statistically significant in our genome‐wide analyses after Bonferroni correction.

### 3.3 Fetal sex determination based on DNA methylation

**FIGURE 3:**
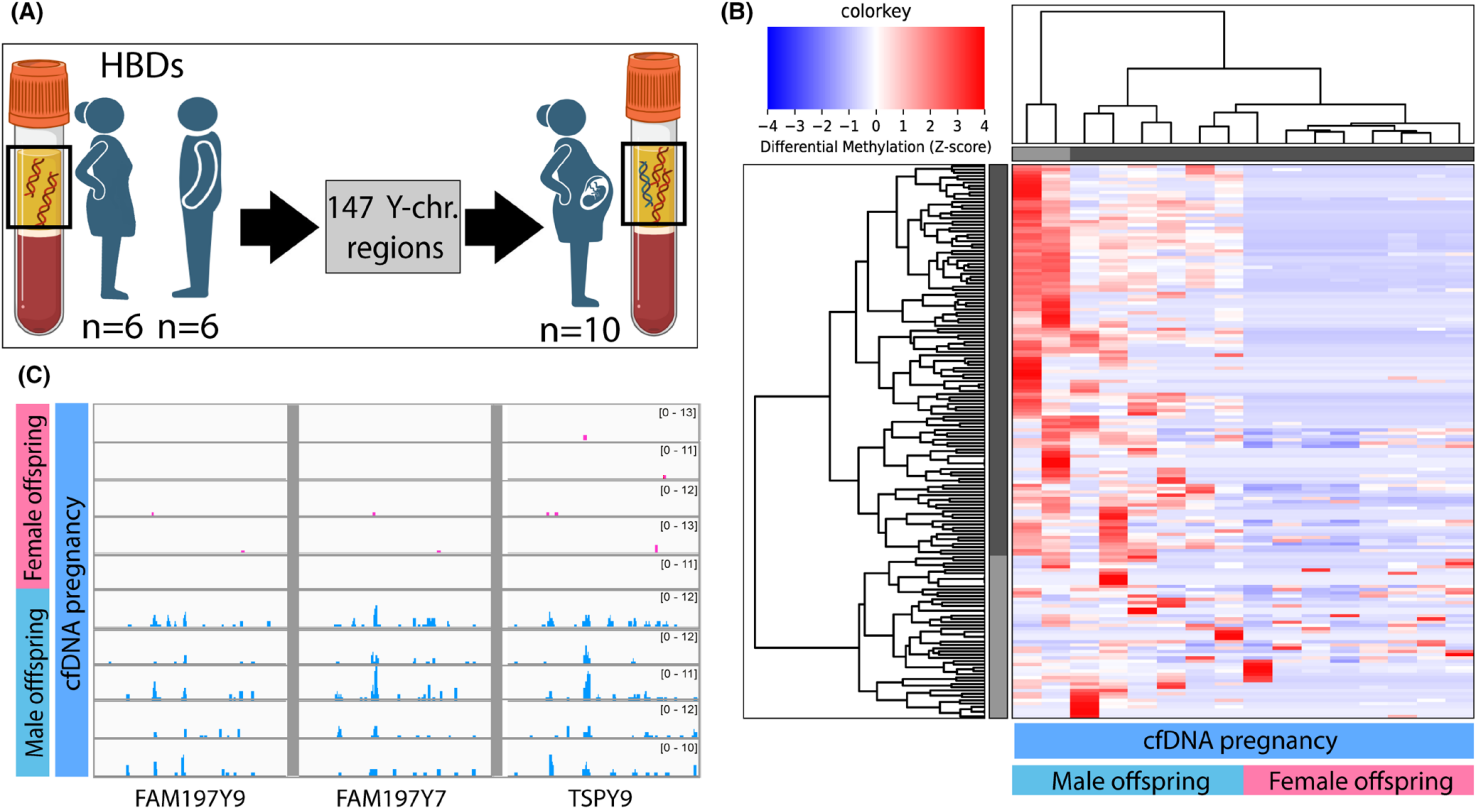
Fetal sex determination in cfDNA from pregnant women. (A) Y‐chromosomal regions containing DNA methylation were identified between male and female healthy blood donors (HBDs). Identified Y‐chromosomal regions were studied in cfDNA from pregnant women. (B) Heatmap visualizing unsupervised hierarchical clustering of cfDNA from pregnant women based on identified Y‐chromosomal regions shows clear clusters of women bearing male offspring (blue bar, left) and female offspring (pink bar, right). Red represents hypermethylation, blue represents hypomethylation. (C) Example gene‐tracks of Y‐chromosomal regions in cfDNA from pregnant women showing methylation only in women bearing male offspring.

To assess the feasibility of determining fetal sex using cfDNA methylation profiles, we first identified 147 Y‐chromosomal regions containing DNA methylation with a FC ≥2 in cfDNA from male (*n* = 6) compared to female HBDs (*n* = 6) (Figure 3A, Table S5). Using identified Y‐chromosomal regions, two clear clusters were identified in maternal cfDNA (Figure 3B). Women bearing boys (*n* = 5) showed hypermethylation at numerous Y‐chromosomal regions as compared to women pregnant with girls (*n* = 5) (Figure 3B,C). Epigenetic marks can thus be used to determine fetal sex in maternal cfDNA. The minimal detection of Y‐chromosomal reads in women pregnant with girls (Figure 3B,C), can be explained by mapping errors caused by large regions displaying high levels of homology between parts of the X‐and Y‐chromosome.

### 3.4 Overlap identified DMRs with buffy coats and novel placental‐specific markers

Not all DMRs identified in maternal cfDNA were detected in placental tissues (Figure 2A,B, Table S3). This could indicate pregnancy‐induced changes in maternal cfDNA from other tissues. During pregnancy, physiological changes result in an increase in white blood cells while simultaneously a change in composition of maternal immune cells occurs.^42^ Since most cfDNA originates from maternal haematopoietic cells,^10^ we hypothesized that pregnancy‐induced physiological immunological changes might explain part of the observed methylation differences between cfDNA from pregnant and nonpregnant women.

**FIGURE 4:**
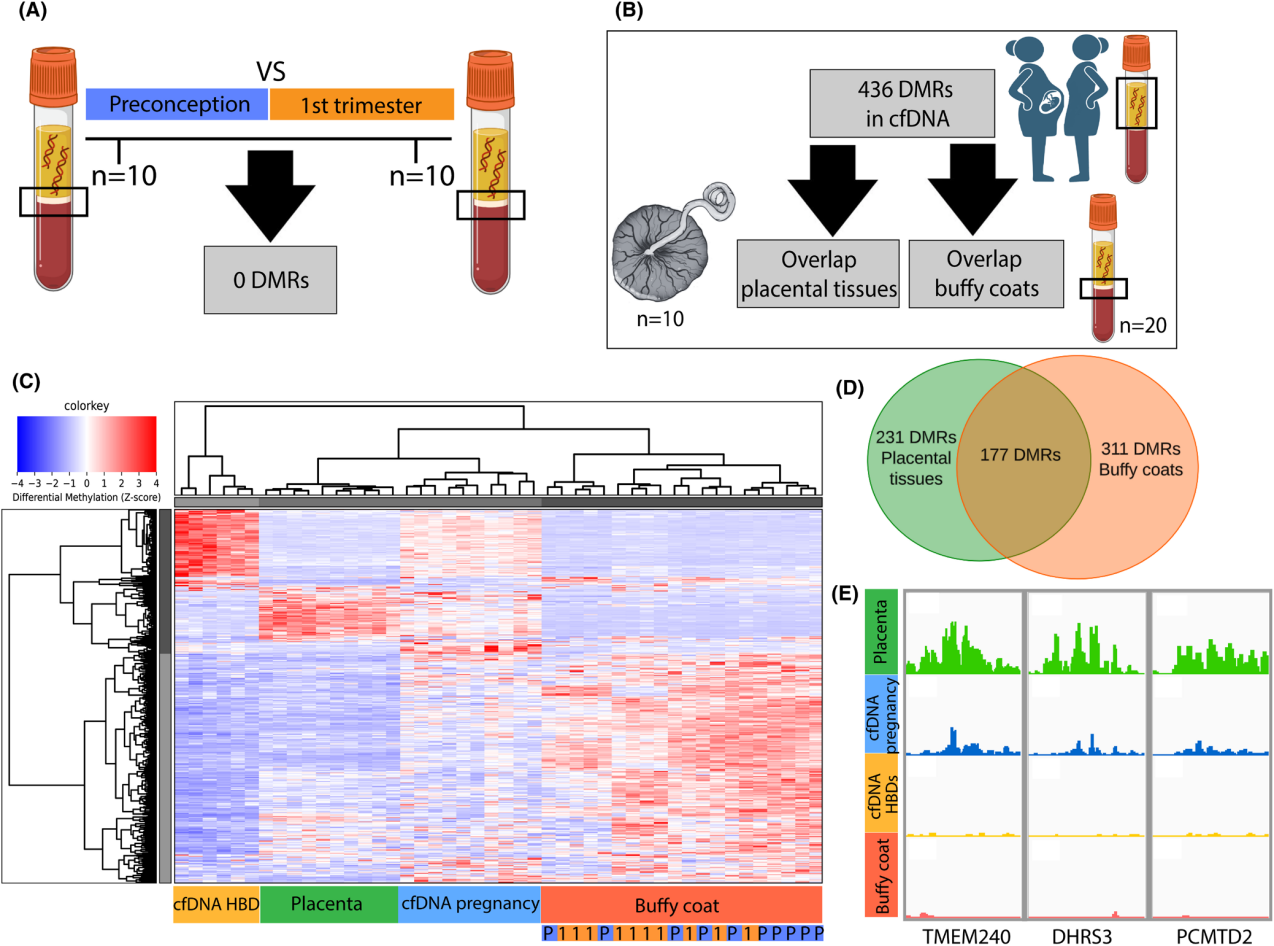
Origin of DMRs identified in cfDNA: Overlap with placental tissues and buffy coats and identification of novel placental‐markers in cfDNA. (A) No DMRs were found between paired buffy coats collected preconception and in the first trimester of pregnancy. (B) Overlap with DNA methylation in placental tissue biopsies and pooled buffy coats was studied for DMRs identified in cfDNA. (C) Heatmap visualizing unsupervised hierarchical clustering for DMRs found between cfDNA from pregnant women and nonpregnant women (HBDs), shows larger overlap of buffy coats with cfDNA from pregnant women as with cfDNA from nonpregnant women. Selected autosomal DMRs were identified with a Fold Change ≥2. Red represents hypermethylation, blue represents hypomethylation. Buffy coats collected during the first trimester pregnancy (P, blue) cluster between buffy coats collected preconceptionally (1, orange). (D) Venn diagram visualizing overlap of DMRs identified in cfDNA from pregnant women compared to nonpregnant women with first trimester placental tissue biopsies and buffy coats. Used cutoff is DMRs present in ≥80% of placental tissue biopsies or buffy coats based on our cumulative methylation score (Table S3). E) Example gene‐tracks of potential placental‐specific DMRs in maternal cfDNA. Gene‐tracks for DMRs with highest fold change between cfDNA from pregnant and nonpregnant women. DMRs are hypermethylated in cfDNA from pregnant women and placental tissue biopsies as compared to cfDNA from nonpregnant women (HBDs). DMRs corresponding to TMEM240, DHRS3 and a CpG island upstream of PCMTD2 show no hypermethylation in buffy coats indicative of a placental‐origin.

To first identify DNA methylation changes in buffy coats related to pregnancy, we compared DNA methylation profiles between paired buffy coats collected preconception (*n* = 10) and in the first trimester of a subsequent pregnancy (*n* = 10) (Figure 4A). Baseline characteristics of included women are depicted in Table S6. In contrast to cfDNA, we identified no DMRs between preconception and first trimester buffy coats.

To further explore possible origins of DMRs identified in cfDNA, next to overlap with placental tissues, we studied overlap of identified DMRs with pooled buffy coats (*n* = 20) (Figure 4B). Surprisingly, the majority of DMRs identified in maternal cfDNA were also present in buffy coats (Figure 4C,D). Based on our cumulative methylation score, 226 (66.9%) of DMRs hypermethylated and 85 (86.7%) of DMRs hypomethylated in maternal cfDNA were respectively also hyper‐or hypomethylated in ≥80% of buffy coat samples (Table S3).

While 231 (53.0%) of DMRs identified in maternal cfDNA were present in ≥80% of placental tissue biopsies, 177 of these were also present in ≥80% of buffy coat samples (Figure 4D, Table S3). These DMRs are therefore not placental‐specific, but could also be of haematopoietic origin. The large overlap of DMRs in maternal cfDNA with DNA methylation of buffy coats might reflect an overall increased immune response during pregnancy.

DMRs in maternal cfDNA that overlap with DNA methylation in placental tissues but not with buffy coats are potential placental‐specific DMRs. We identified 16 DMRs that were hypermethylated in ≥80% of both maternal cfDNA and placental tissue samples, while hypomethylated in both ≥80% of HBD cfDNA and buffy coat samples (Table S3). These DMRs are therefore likely to represent placental‐specific markers in cfDNA. To illustrate, Figure 4E shows gene tracks for 3 DMRs with the highest FC between cfDNA from pregnant and nonpregnant women. The three top‐ranked DMRs correspond to *TMEM240* (FC 10.2), *DHRS3* (FC 7.6) and a CpG island 40 kb upstream of *PCMTD2* (FC 6.5).

## 4 DISCUSSION

We applied MeD‐seq to investigate the genome‐wide impact of first trimester pregnancy on the maternal plasma‐derived cfDNA methylome. We identified numerous DMRs between cfDNA from pregnant and nonpregnant women. Next, we confirmed identification of placental‐specific methylation marks in maternal cfDNA by showing hypermethylation of the *RASSF1* promoter—a known placental‐specific marker—and the possibility to determine fetal sex based on Y‐chromosomal DNA methylation. Moreover, we identified novel potential placental‐specific methylation marks in maternal cfDNA, such as hypermethylated DMRs in *TMEM240*, *DHRS3* and a CpG island upstream of *PCMTD2*, that were more robust as compared to *RASSF1* hypermethylation.

Although the majority of DMRs identified in maternal cfDNA overlapped with placental DNA methylation, most DMRs also overlapped with DNA methylation of buffy coats. Our results suggest a more prominent immune‐cell type(s)‐related contribution to DMRs in cfDNA from pregnant as compared to nonpregnant women, and thus both a placental and immune‐cell contribution to the pregnancy‐specific cfDNA methylation signature. This is in line with other studies that recently found that the increased cfDNA levels in patients with several types of cancers are predominantly caused by increased levels of leukocyte‐derived cfDNA instead of tumour‐derived cfDNA,^43^, ^44^ as is also found for patients with acute pancreatitis or sepsis. Our findings therefore suggest that the cfDNA methylation profile reflects an overall increased immune response during pregnancy.^10^, ^45^ Although the presence of placental‐originated cfDNA fragments influences the maternal cfDNA methylome, it is important to acknowledge the large contribution of other maternal tissues to total maternal cfDNA when interpreting methylation changes related to pregnancy.

DMRs in maternal cfDNA that were not shared with either placental tissues or buffy coats could be attributable to physiological pregnancy‐related changes in other (high‐turnover) maternal tissues that contribute to cfDNA, such as liver or endothelium.^10^ Another explanation could be a different contribution of haematopoietic and placental cell types to buffy coats or tissue biopsies as compared to their contribution to cfDNA. For example, in placental tissue biopsies, DNA methylation reflects the average methylation of all cells in the selected sample, while for cfDNA the contribution of different placental cell types could be influenced by apoptosis rate and anatomical position in relation to the maternal blood stream.

Although fetal sex can already be determined in maternal cfDNA based on the presence of Y‐chromosomal genetic material,^2^ we were able to establish the fetal sex by combining numerous methylated Y‐chromosomal regions in maternal cfDNA. In the future, we aim to investigate the use of DNA methylation data from all chromosomes for the detection of aneuploidies as previously shown by others.^15^, ^16^, ^21^, ^22^, ^23^ The potential added value of combining genetic and epigenetic information in NIPT warrants further study.

MeD‐seq can be used to discover genome‐wide DMRs directly in cfDNA, instead of using differences between whole blood and placental tissues. This may more reflect the turnover of specific cell types and can lead to additional markers relevant for cfDNA applications in pregnancy. For example, the previously mentioned hypermethylation of DMRs in *TMEM240*, *DHRS3* and a CpG island upstream of *PCMTD2* were more robust in cfDNA of pregnant compared to nonpregnant women, as compared to *RASSF1* hypermethylation. Application of the UCSC database shows enrichment for regulatory regions (ReMap database) for two of our DMRs (*DHRS3* and *PCMTD2*), possibly related to a functional role for DNA methylation in gene regulation of nearby genes. The three top‐ranked DMRs show enrichment for binding of numerous proteins, mainly including BRD4 which is involved in regulating cell differentiation, and ESR1 and CTCF, which play a role in transcription regulation. Additionally, comparable to hypermethylation of *RASSF1*, hypermethylation of *TMEM240* and *DHRS3* have been described in several types of cancers, and these genes have been proposed as tumour suppressor genes.^46^, ^47^, ^48^ Similarities in DNA methylation between the placenta and cancers are well known and are suggested to be involved in their mutual properties such as proliferation, cell invasion and immune modulation.^49^

A previous study identified differentially methylated CpGs between cfDNA from pregnant and nonpregnant women.^30^ They found 8277 differently methylated CpGs, of which 1704 CpGs, corresponding to 501 genes, were hypermethylated in cfDNA from pregnant compared to nonpregnant women, as well as in chorionic villus samples compared to maternal leukocytes. Although we focused on DMRs instead of individual CpGs, 11 of these identified genes overlapped with genes corresponding to our identified DMRs, of which 6 of the previously identified CpGs were captured by our DMRs: *DCAF10*, *DENND2D*, *OSR2*, *RNF126P1*, *TMEM17* and *TMEM233* (Figure S2). These DMRs are therefore also likely robust placental‐specific DNA methylation markers in maternal cfDNA.

In contrast to this previous study, most DMRs identified in our study were hypermethylated instead of hypomethylated in cfDNA from pregnant as compared to nonpregnant women. This bias towards hypermethylated regions when using MeD‐seq could be explained by technological considerations combined with a ‘diluted’ placental signal within the total pool of maternal cfDNA. For example, for regions highly methylated in blood cells and thus in cfDNA, but hypomethylated in placental tissues, the relative decrease in methylation of maternal cfDNA caused by hypomethylated placental‐originated cfDNA fragments will often be limited and remain undetected.

The relatively low contribution of placental‐derived cfDNA remains a major challenge when using cfDNA as a proxy for placental DNA.^11^, ^12^ In future studies, fragment size of cfDNA may be used to enrich for placental‐originated cfDNA, since placental‐originated cfDNA fragments are shorter than maternal cfDNA fragments.^50^ Combining DNA methylation analyses with other cfDNA features, referred to as ‘fragmentomics’, could improve tissue of origin identification and analyses.^50^, ^51^ Importantly, the contribution of different placental cell types (e.g. syncytiotrophoblast, cytotrophoblast and extravillous trophoblast) to cfDNA is currently unclear and probably also changes during gestation, driven by developmental changes in the placenta composition itself, as is observed for cell‐free RNA.^52^ Therefore, future studies should strive to establish DNA methylation profiles for specific placental cell type as reference.

Recent studies have shown promising results for the use of the cfDNA methylome as predictor of obstetric complications, such as pre‐eclampsia.^32^, ^33^ More research is warranted to establish robust, easy to use biomarkers for obstetric health based on the cfDNA methylome, and to investigate whether these DNA methylation biomarkers could for example be incorporated into the early prenatal genetic screening.^53^ Of note, cfDNA methylation differences for biomarker development do not have to originate from placental cfDNA, but could thus also originate from maternal tissues such as haematopoietic cells.

This study is one of the first to study genome‐wide methylation of cfDNA in pregnant women. As compared to other techniques, MeD‐seq could be a less costly and easier to perform method for low concentration cfDNA methylation profiling while ensuring a high genome‐wide coverage. However, the limitations of this pilot study are that we did not correct for potential confounders such as age, ethnicity and smoking which can impact DNA methylation.^54^, ^55^, ^56^ For HBDs and placental tissues, we had no access to detailed participant's characteristics. Furthermore, MeD‐seq has a bias towards hypermethylated regions, and the highly fragmented nature of cfDNA may influence the coverage of the genome. Lastly, differences in placental cell type contributions in tissue biopsies and cfDNA could have distorted our results. Future studies should therefore aim to establish reference methylation profiles for different placental cell types.

## 5 CONCLUSION

Research into the cfDNA methylome could allow for cfDNA applications beyond current practice; however, exploring the epigenetic landscape of cfDNA during pregnancy is still in its infancy. This pilot study revealed feasibility of genome‐wide methylation profiling of maternal cfDNA using the MeD‐seq technology. As a proof‐of‐concept, we showed that genome‐wide placental DNA methylation marks can be identified in first trimester maternal cfDNA, and fetal sex can be determined based on Y‐chromosomal DNA methylation. Moreover, our results indicate not only a placental‐origin but also a significant immune‐cell contribution to the pregnancy‐specific cfDNA methylation signature. The added value of epigenome‐wide analyses of cfDNA using MeD‐seq in the current practice of prenatal genetic testing and in relation to obstetric complications and gestational age warrants further study.

## AUTHOR CONTRIBUTIONS

MMV, RGB, OJMS, RPMST, JG and SS were involved in the study design. MMV, RGB and JBB were involved in data analysis. MMV wrote the first draft of the manuscript. RST is the principal investigator of the Rotterdam Periconception cohort. MV and SS were involved in acquiring placental tissues and MMV and LEM were involved in processing of placental tissues. LM performed histopathologic examination of all placental tissues. RGB, JBB and JG are co‐founders of the MeD‐seq technology. All authors were involved in co‐writing and critical discussion of the manuscript, and all authors read and approved the final manuscript.

## FUNDING INFORMATION

This research was funded by the Department of Obstetrics and Gynaecology and the Department of Developmental Biology of the Erasmus MC, University Medical Center, Rotterdam, The Netherlands.

## CONFLICT OF INTEREST STATEMENT

The authors report there are no competing interests to declare except for RGB, JBB and JG, who report being shareholder in Methylomics B.V., a commercial company that applies MeD‐seq.

## Supporting information


Figures S1–S2.

Tables S1–S6.

## Data Availability

The MeD‐seq datasets supporting the conclusions of this article are available in the Sequence Read Archive (SRA) repository at the National Center for Biotechnology Information, with accession number PRJNA1108949 via https://www.ncbi.nlm.nih.gov/sra/?term=PRJNA1108949.

## References

[1] Wilson R , Gagnon A , Audibert F , Campagnolo C , Carroll J , COMMITTEE. G . Prenatal diagnosis procedures and techniques to obtain a diagnostic fetal specimen or tissue: maternal and fetal risks and benefits. J Obstet Gynaecol Can. 2015;37(7):656‐668. 26366824 10.1016/S1701-2163(15)30205-X

[2] Mackie F , Hemming K , Allen S , Morris R , Kilby M . The accuracy of cell‐free fetal DNA‐based non‐invasive prenatal testing in singleton pregnancies: a systematic review and bivariate meta‐analysis. BJOG. 2017;124(1):32‐46. 27245374 10.1111/1471-0528.14050

[3] Hui W , Chiu R . Noninvasive prenatal testing beyond genomic analysis: what the future holds. Curr Opin Obstet Gynecol. 2016;28(2):105‐110. 26866842 10.1097/GCO.0000000000000252

[4] Moore L , Le T , Fan G . DNA methylation and its basic function. Neuropsychopharmacology. 2012;38(1):23‐38. 22781841 10.1038/npp.2012.112 PMC3521964

[5] Januar V , Desoye G , Novakovic B , Cvitic S , Saffery R . Epigenetic regulation of human placental function and pregnancy outcome: considerations for causal inference. Am J Obstet Gynecol. 2015;213(4 Suppl):S182‐S196. 26428498 10.1016/j.ajog.2015.07.011

[6] Nelissen E , Montfoort van A , Dumoulin J , Evers J . Epigenetics and the placenta. Hum Reprod Update. 2011;17(3):397‐417. 20959349 10.1093/humupd/dmq052

[7] Lee Y , Choufani S , Weksberg R , et al. Placental epigenetic clocks: estimating gestational age using placental DNA methylation levels. Aging (Albany NY). 2019;11(12):4238‐4253. 31235674 10.18632/aging.102049 PMC6628997

[8] Cruz de OJ , Conceição I , Tosatti J , Gomes K , Luizon M . Global DNA methylation in placental tissues from pregnant with preeclampsia: a systematic review and pathway analysis. Placenta. 2020;101:97‐107. 32942147 10.1016/j.placenta.2020.09.004

[9] Cirkovic A , Garovic V , Lazovic J , et al. Systematic review supports the role of DNA methylation in the pathophysiology of preeclampsia: a call for analytical and methodological standardization. Biol Sex Differ. 2020;11(1):36. 32631423 10.1186/s13293-020-00313-8 PMC7336649

[10] Moss J , Magenheim J , Neiman D , et al. Comprehensive human cell‐type methylation atlas reveals origins of circulating cell‐free DNA in health and disease. Nat Commun. 2018;9(1):5068. 30498206 10.1038/s41467-018-07466-6 PMC6265251

[11] Hestand M , Bessem M , van Rijn P , et al. Fetal fraction evaluation in non‐invasive prenatal screening (NIPS). Eur J Hum Genet. 2019;27(2):198‐202. 30254213 10.1038/s41431-018-0271-7 PMC6336813

[12] Deng C , Liu S . Factors affecting the fetal fraction in noninvasive prenatal screening: a review. Front Pediatr. 2022;10:812781. 35155308 10.3389/fped.2022.812781 PMC8829468

[13] Oberhofer A , Bronkhorst A , Uhlig C , Ungerer V , Holdenrieder S . Tracing the origin of cell‐free DNA molecules through tissue‐specific epigenetic signatures. Diagnostics (Basel). 2022;12(8):1834. 36010184 10.3390/diagnostics12081834 PMC9406971

[14] Papageorgiou E , Fiegler H , Rakyan V , et al. Sites of differential DNA methylation between placenta and peripheral blood: molecular markers for noninvasive prenatal diagnosis of aneuploidies. Am J Pathol. 2009;174(5):1609‐1618. 19349366 10.2353/ajpath.2009.081038 PMC2671250

[15] Keravnou A , Ioannides M , Loizides C , et al. MeDIP combined with in‐solution targeted enrichment followed by NGS: inter‐individual methylation variability of fetal‐specific biomarkers and their implementation in a proof of concept study for NIPT. PLoS One. 2018;13(6):e0199010. 29889893 10.1371/journal.pone.0199010 PMC5995407

[16] Tong Y , Jin S , Chiu R , et al. Noninvasive prenatal detection of trisomy 21 by an epigenetic‐genetic chromosome‐dosage approach. Clin Chem. 2010;56(1):90‐98. 19850629 10.1373/clinchem.2009.134114

[17] Chim S , Jin S , Lee T , et al. Systematic search for placental DNA‐methylation markers on chromosome 21: toward a maternal plasma‐based epigenetic test for fetal trisomy 21. Clin Chem. 2008;54(3):500‐511. 18202156 10.1373/clinchem.2007.098731

[18] Chu T , Burke B , Bunce K , Surti U , Allen Hogge W , Peters D . A microarray‐based approach for the identification of epigenetic biomarkers for the noninvasive diagnosis of fetal disease. Prenat Diagn. 2009;29(11):1020‐1030. 19650061 10.1002/pd.2335

[19] Bunce K , Chu T , Surti U , Hogge W , Peters D . Discovery of epigenetic biomarkers for the noninvasive diagnosis of fetal disease. Prenat Diagn. 2012;32(6):542‐549. 22495992 10.1002/pd.3853 PMC4308692

[20] Ioannides M , Papageorgiou E , Keravnou A , et al. Inter‐individual methylation variability in differentially methylated regions between maternal whole blood and first trimester CVS. Mol Cytogenet. 2014;7(1):73. 25426166 10.1186/s13039-014-0073-8 PMC4243368

[21] Gordevičius J , Narmontė M , Gibas P , et al. Identification of fetal unmodified and 5‐hydroxymethylated CG sites in maternal cell‐free DNA for non‐invasive prenatal testing. Clin Epigenetics. 2020;12(1):153. 33081811 10.1186/s13148-020-00938-x PMC7574562

[22] Papageorgiou E , Karagrigoriou A , Tsaliki E , Velissariou V , Carter N , Patsalis P . Fetal‐specific DNA methylation ratio permits noninvasive prenatal diagnosis of trisomy 21. Nat Med. 2011;17(4):510‐513. 21378977 10.1038/nm.2312 PMC3977039

[23] Coppedè F , Bhaduri U , Stoccoro A , Nicolì V , Di Venere E , Merla G . DNA methylation in the fields of prenatal diagnosis and early detection of cancers. Int J Mol Sci. 2023;24(14):11715. 37511475 10.3390/ijms241411715 PMC10380460

[24] Xiang Y , Zhang J , Li Q , et al. DNA methylome profiling of maternal peripheral blood and placentas reveal potential fetal DNA markers for non‐invasive prenatal testing. Mol Hum Reprod. 2014;20(9):875‐884. 24996894 10.1093/molehr/gau048

[25] Ou X , Wang H , Qu D , Chen Y , Gao J , Sun H . Epigenome‐wide DNA methylation assay reveals placental epigenetic markers for noninvasive fetal single‐nucleotide polymorphism genotyping in maternal plasma. Transfusion. 2014;54(10):2523‐2533. 24749853 10.1111/trf.12659

[26] Keravnou A , Ioannides M , Tsangaras K , et al. Whole‐genome fetal and maternal DNA methylation analysis using MeDIP‐NGS for the identification of differentially methylated regions. Genet Res (Camb). 2016;98:e15. 27834155 10.1017/S0016672316000136 PMC6865150

[27] Jensen T , Kim S , Zhu Z , et al. Whole genome bisulfite sequencing of cell‐free DNA and its cellular contributors uncovers placenta hypomethylated domains. Genome Biol. 2015;16(1):78. 25886572 10.1186/s13059-015-0645-x PMC4427941

[28] Lun F , Chiu R , Sun K , et al. Noninvasive prenatal methylomic analysis by genomewide bisulfite sequencing of maternal plasma DNA. Clin Chem. 2013;59(11):1583‐1594. 23857673 10.1373/clinchem.2013.212274

[29] Del Vecchio G , Li Q , Li W , et al. Cell‐free DNA methylation and transcriptomic signature prediction of pregnancies with adverse outcomes. Epigenetics. 2021;16(6):642‐661. 33045922 10.1080/15592294.2020.1816774 PMC8143248

[30] Chu T , Shaw P , McClain L , Simhan H , Peters D . High‐resolution epigenomic liquid biopsy for noninvasive phenotyping in pregnancy. Prenat Diagn. 2021;41(1):61‐69. 33002217 10.1002/pd.5833

[31] Spinelli M , Zdanowicz J , Keller I , et al. Hypertensive disorders of pregnancy share common cfDNA methylation profiles. Sci Rep. 2022;12(1):19837. 36400896 10.1038/s41598-022-24348-6 PMC9674847

[32] De Borre M , Che H , Yu Q , et al. Cell‐free DNA methylome analysis for early preeclampsia prediction. Nat Med. 2023;29(9):2206‐2215. 37640858 10.1038/s41591-023-02510-5

[33] He W , Zhang Y , Wu K , et al. Epigenetic phenotype of plasma cell‐free DNA in the prediction of early‐onset preeclampsia. J Obstet Gynaecol. 2023;43(2):2282100. 38038254 10.1080/01443615.2023.2282100

[34] Deger T , Boers R , de Weerd V , et al. High‐throughput and affordable genome‐wide methylation profiling of circulating cell‐free DNA by methylated DNA sequencing (MeD‐seq) of LpnPI digested fragments. Clin Epigenetics. 2021;13(1):196. 34670587 10.1186/s13148-021-01177-4 PMC8529776

[35] Boers R , Boers J , Hoon de B , et al. Genome‐wide DNA methylation profiling using the methylation‐dependent restriction enzyme LpnPI. Genome Res. 2018;28(1):88‐99. 29222086 10.1101/gr.222885.117 PMC5749185

[36] Rousian M , Schoenmakers S , Eggink A , et al. Cohort profile update: the Rotterdam periconceptional cohort and embryonic and fetal measurements using 3D ultrasound and virtual reality techniques. Int J Epidemiol. 2021;50:1426‐1427l. 34097026 10.1093/ije/dyab030 PMC8580268

[37] Steegers‐Theunissen R , Verheijden‐Paulissen J , van Uitert E , et al. Cohort profile: the Rotterdam periconceptional cohort (predict study). Int J Epidemiol. 2016;45(2):374‐381. 26224071 10.1093/ije/dyv147

[38] van Dessel L , Beije N , Helmijr J , et al. Application of circulating tumor DNA in prospective clinical oncology trials ‐ standardization of preanalytical conditions. Mol Oncol. 2017;11(3):295‐304. 28164427 10.1002/1878-0261.12037 PMC5527445

[39] Bos M , Verhoeff S , Oosting S , et al. Methylated cell‐free DNA sequencing (MeD‐seq) of LpnPI digested fragments to identify early progression in metastatic renal cell carcinoma patients on watchful waiting. Cancers (Basel). 2023;15(5):1374. 36900167 10.3390/cancers15051374 PMC10000042

[40] Zejskova L , Jancuskova T , Kotlabova K , Doucha J , Hromadnikova I . Feasibility of fetal‐derived hypermethylated RASSF1A sequence quantification in maternal plasma — next step toward reliable non‐invasive prenatal diagnostics. Exp Mol Pathol. 2010;89(3):241‐247. 20868679 10.1016/j.yexmp.2010.09.002

[41] Chan K , Ding C , Gerovassili A , et al. Hypermethylated RASSF1A in maternal plasma: a universal fetal DNA marker that improves the reliability of noninvasive prenatal diagnosis. Clin Chem. 2006;52(12):2211‐2218. 17068167 10.1373/clinchem.2006.074997

[42] Abu‐Raya B , Michalski C , Sadarangani M , Lavoie P . Maternal immunological adaptation during Normal pregnancy. Front Immunol. 2020;11:575197. 33133091 10.3389/fimmu.2020.575197 PMC7579415

[43] Mattox A , Douville C , Wang Y , et al. The origin of highly elevated cell‐free DNA in healthy individuals and patients with pancreatic, colorectal, lung, or ovarian cancer. Cancer Discov. 2023;13(10):2166‐2179. 37565753 10.1158/2159-8290.CD-21-1252 PMC10592331

[44] Thierry A , Pisareva E . A new paradigm of the origins of circulating DNA in patients with cancer. Cancer Discov. 2023;13(10):2122‐2124. 37794839 10.1158/2159-8290.CD-23-0824

[45] Liu X , Ren J , Luo N , et al. Comprehensive DNA methylation analysis of tissue of origin of plasma cell‐free DNA by methylated CpG tandem amplification and sequencing (MCTA‐Seq). Clin Epigenetics. 2019;11(1):93. 31234922 10.1186/s13148-019-0689-y PMC6591962

[46] Lin R , Su C , Lin S , et al. Hypermethylation of TMEM240 predicts poor hormone therapy response and disease progression in breast cancer. Mol Med. 2022;28(1):67. 35715741 10.1186/s10020-022-00474-9 PMC9204905

[47] Chang S , Liew P , Ansar M , et al. Hypermethylation and decreased expression of TMEM240 are potential early‐onset biomarkers for colorectal cancer detection, poor prognosis, and early recurrence prediction. Clin Epigenetics. 2020;12(1):67. 32398064 10.1186/s13148-020-00855-z PMC7218647

[48] Sumei S , Xiangyun K , Fenrong C , et al. Hypermethylation of DHRS3 as a novel tumor suppressor involved in tumor growth and prognosis in gastric cancer. Front Cell Dev Biol. 2021;9:624871. 33553182 10.3389/fcell.2021.624871 PMC7859350

[49] Costanzo V , Bardelli A , Siena S , Abrignani S . Exploring the links between cancer and placenta development. Open Biol. 2018;8(6):180081. 29950452 10.1098/rsob.180081 PMC6030113

[50] Lo Y , Han D , Jiang P , Chiu R . Epigenetics, fragmentomics, and topology of cell‐free DNA in liquid biopsies. Science. 2021;372(6538):eaaw3616. 33833097 10.1126/science.aaw3616

[51] Mouliere F . A hitchhiker's guide to cell‐free DNA biology. Neurooncol Adv. 2022;4(Suppl 2):ii6‐ii14. 36380865 10.1093/noajnl/vdac066 PMC9650475

[52] Tsang J , Vong J , Ji L , et al. Integrative single‐cell and cell‐free plasma RNA transcriptomics elucidates placental cellular dynamics. Proc Natl Acad Sci USA. 2017;114(37):E7786‐E7795. 28830992 10.1073/pnas.1710470114 PMC5604038

[53] Aerden M , De Borre M , Thienpont B . Cell‐free DNA methylation‐based preeclampsia prediction: a journey to improve maternal health. Prenat Diagn. 2024;44(4):418‐421. 38047711 10.1002/pd.6478

[54] Jung M , Pfeifer G . Aging and DNA methylation. BMC Biol. 2015;13:7. 25637097 10.1186/s12915-015-0118-4 PMC4311512

[55] Elliott H , Burrows K , Min J , et al. Characterisation of ethnic differences in DNA methylation between UK‐resident south Asians and Europeans. Clin Epigenetics. 2022;14(1):130. 36243740 10.1186/s13148-022-01351-2 PMC9571473

[56] Fragou D , Pakkidi E , Aschner M , Samanidou V , Kovatsi L . Smoking and DNA methylation: correlation of methylation with smoking behavior and association with diseases and fetus development following prenatal exposure. Food Chem Toxicol. 2019;129:312‐327. 31063835 10.1016/j.fct.2019.04.059

