# Supplementary material for: Genome‐wide methylation profiling of maternal cell‐free DNA using methylated DNA sequencing (MeD‐seq) indicates a placental and immune‐cell signature
Source: Eur J Clin Invest. 2024 Nov 26;55(3):e14363. doi: 10.1111/eci.14363 (PMC11810559; doi:10.1111/eci.14363)
Supplement: Supplementary file 1 — Figures S1–S2. [file ECI-55-e14363-s002.zip › FiguresS1-S2_Caption.docx]

Figure S1: Heatmap visualizing overlap between cfDNA from male (M) and female (F) non-pregnant healthy blood donors (HBDs) compared to cfDNA from pregnant women, based on DMRs identified between pregnant women (P) and non-pregnant female HBDs. Red represents hypermethylation, blue represents hypomethylation.

Figure S2: Gene-tracks for DMRs between cfDNA from pregnant women and non-pregnant female healthy blood donors (HBDs) that overlap with CpGs previously identified by Chu et al^30^
