# Supplementary figures and images for: Genome‐wide methylation profiling of maternal cell‐free DNA using methylated DNA sequencing (MeD‐seq) indicates a placental and immune‐cell signature
Source: Eur J Clin Invest. 2024 Nov 26;55(3):e14363. doi: 10.1111/eci.14363 (PMC11810559; doi:10.1111/eci.14363)

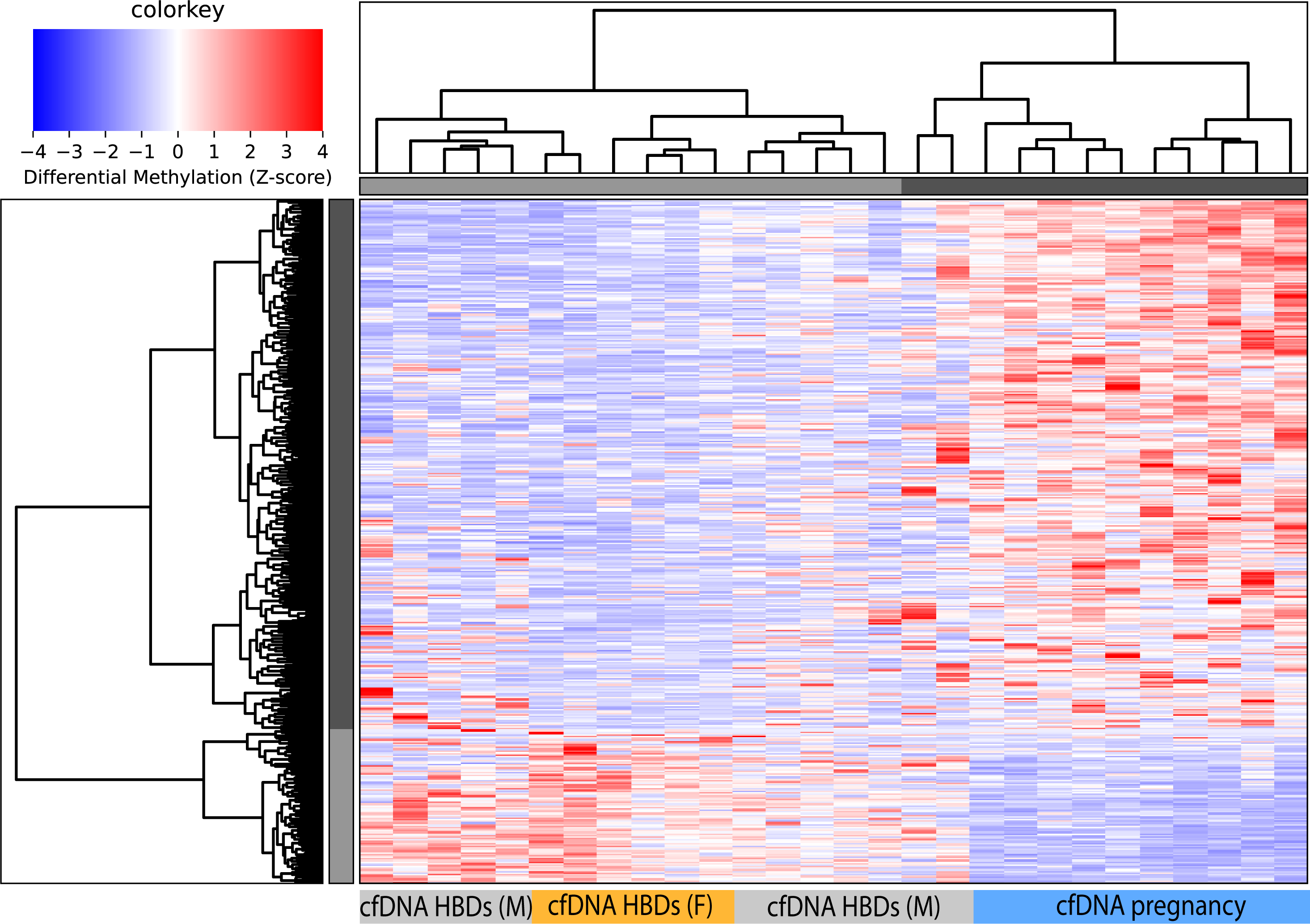

Supplement: Supplementary file 1 — Figures S1–S2. [file ECI-55-e14363-s002.zip › Figure_S1.tif]

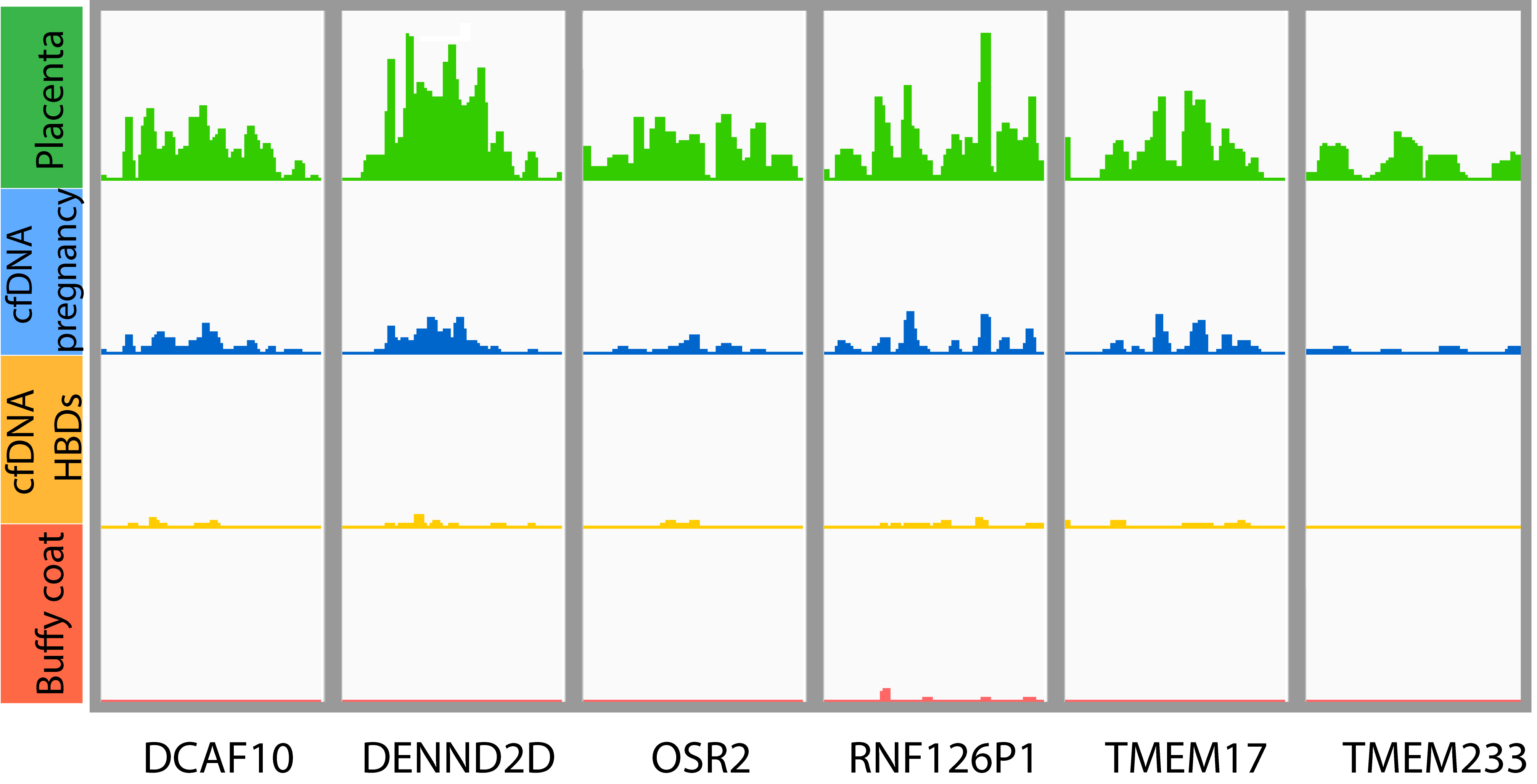

Supplement: Supplementary file 1 — Figures S1–S2. [file ECI-55-e14363-s002.zip › Figure_S2.tif]
